# Retraction Note: Mst1 overexpression combined with Yap knockdown augments thyroid carcinoma apoptosis via promoting MIEF1-related mitochondrial fission and activating the JNK pathway

**DOI:** 10.1186/s12935-023-02921-x

**Published:** 2023-04-17

**Authors:** Xiaoli Zhang, Fei Li, Yeqing Cui, Shuang Liu, Haichen Sun

**Affiliations:** Department of General Surgery, Xuanwu Hospital, Capital Medical University, #45, Chang Chun Street, Beijing 100053, China

**Retraction Note: Cancer Cell Int (2019) 19:143**

10.1186/s12935-019-0860-8

The Editors-in-Chief have retracted this article. After publication, concerns were raised regarding image similarities between this article and several other articles. Specifically:

- Figure 1a GAPDH blot appears highly similar to Fig. 7a t-JNK in [1], which was under consideration within a similar time frame as this article.
- Figure 2a Cyclin D1 blot appears highly similar to Fig. 3e CIV-II in [1].
- Figure 2a CDK4 blot appears highly similar to Fig. S1e Bcl-2 and in this article, Fig. 3b CIV-II in [2] and Fig. 2d CXCR7 (flipped vertically) in [3].
- In Fig. 2g, the Ctrl and Ad-Mst1 images appear to share some highly similar features; the Ad-Mst1 image also appears to contain some repetitive elements.
- Figure 3 h Bcl2 blot appears highly similar to Fig. 3b CIII-core2 in [2].
- Figure 3 g has very unusual shapes for flow cytometry plots.
- Figure 4a Ctrl and Ad-Mst1 + sh-Yap images appear highly similar to Fig. 4a Melatonin and TNFα images (rotated), respectively, in [2].
- Figure 4c Mff blot appears highly similar to Fig. 3b CII-30 (flipped horizontally) in [2].
- The same Caspase-9 western blots appear to be presented in Fig. 3 h and S1e.
- Figure 4c Drp1 blot appears highly similar to Fig. 2d CyclinD1 (flipped horizontally) in [1].

The Editors-in-Chief therefore no longer have confidence in the presented data.

None of the authors have responded to any correspondence from the editor or publisher about this retraction.
